# Dexamethasone‐Mediated Regulation of CYP3A4 and UGTs in Human Hepatoma HuH‐7 Cells

**DOI:** 10.1111/fcp.70089

**Published:** 2026-04-21

**Authors:** Hana Yu, Song Hee Lee, Ji Hyeon Kim, Seung Jin Kim, Hee Eun Kang

**Affiliations:** ^1^ College of Pharmacy and Integrated Research Institute of Pharmaceutical Sciences The Catholic University of Korea Bucheon South Korea

**Keywords:** constitutive androstane receptor (CAR), cytochrome P450 (CYP), dexamethasone (DEX), glucocorticoid receptor (GR), HuH‐7 cells, pregnane X receptor (PXR), uridine 5′‐diphospho‐glucuronosyltransferase (UGT)

## Abstract

**Background:**

The application of human hepatic cell lines to early drug discovery and development instead of human primary hepatocytes (HPHs) has been limited because of the low level of drug‐metabolizing enzymes (DMEs).

**Objective:**

The study aimed to evaluate the effects of dexamethasone (DEX) treatment on DME expression, activities, and regulation in HuH‐7 hepatoma cells.

**Methods:**

Expression and transcriptional regulation of major CYPs and UGTs in HuH‐7 cells was evaluated by immunoblotting, probe substrate assays, and treatments with nuclear receptor agonists, including DEX, and antagonists.

**Results:**

DEX increased the expression and activity of cytochrome P450 (CYP) 3A4, uridine 5′‐diphospho‐glucuronosyltransferase (UGT) 1A1, and UGT2B7 but had minimal effects on CYP1A2, CYP2B6, or CYP2C9. These augmented activities of CYP3A4, UGT1A1, and UGT2B7 were concentration‐dependently inhibited by their corresponding selective inhibitors. DEX‐induced upregulation of CYP3A4 protein expression was abolished by co‐treatment with the glucocorticoid receptor (GR) inhibitor, but not by co‐treatment with the pregnane X receptor (PXR) inhibitor. However, treatment with PXR or constitutive androstane receptor (CAR) agonist did not lead to transcriptional activation of CYP3A4 and 2B6. Only CYP1A1 was transactivated by an aryl hydrocarbon receptor (AhR) ligand.

**Conclusion:**

Our results suggest that the activities of some major DMEs (CYP3A4, UGT1A1, and UGT2B7) in HuH‐7 cells are promoted by DEX treatment possibly through GR activation. However, unlike HPHs, HuH‐7 cells fail to show transcriptional regulation of DMEs by PXR or CAR, which limits their suitability for evaluating DME induction potential of investigational drugs.

## 1 Introduction

Human hepatoma cell lines provide a cost‐effective and easily maintained alternative to human primary hepatocytes (HPHs), although the loss of several essential hepatocyte functions limits their utility [1, 2, 3, 4, 5]. For instance, the HepG2 cell line exhibits numerous hepatic functions and a high level of phase II enzymes but demonstrates a low level of cytochrome P450 (CYP) enzymes and a lack of responsiveness to the inducers. In contrast, when properly differentiated, HepaRG cells express high and stable levels of major drug‐metabolizing enzymes (DMEs), including CYP1A2, 2B6, 2C9, 2E1, 3A4, and conjugating enzymes, and display inducibility comparable to primary human hepatocytes, making them a reliable in vitro model for human hepatic drug metabolism and detoxification studies [6, 7].

HuH‐7 is an immortal hepatoma cell line [8], taken from the hepatoma tissue of a Japanese male. It exhibits mRNA expression patterns of hepatic DMEs and transporters that closely resemble those of HPHs, more so than the commonly used HepG2 cells [2]. Hepatobiliary transporters exist in HuH‐7 cells, although their expression and function are not as high as in HPHs [9, 10, 11]. Interestingly, keeping HuH‐7 cells confluent over several weeks leads to transcriptional induction of various DMEs and transporters, along with their regulators such as constitutive androstane receptor (CAR), pregnane X receptor (PXR), and retinoid X receptor (RXR) [12]. In addition, HuH‐7 cells exhibit a morphology that resembles hepatocytes, with structures including bile canaliculi following long‐term culture. Interestingly, the functions of some ATP‐binding cassette (ABC) transporters, including bile salt export pump (BSEP), multidrug resistance protein 1 (MDR1), and multidrug resistance‐associated protein 2 (MRP2) can be improved in confluent HuH‐7 cells by modifying culture conditions, such as through dexamethasone (DEX) supplementation and Matrigel overlay [13]. Dimethyl sulfoxide (DMSO) has also been found to facilitate the expression and activity of the key bile acid‐metabolizing enzymes and ‐transporters in DEX‐supplemented HuH‐7 cells [14].

Cultures of HPHs or HepaRG cells typically need supplementation with glucocorticoids, such as DEX to enhance the organization of the cytoskeletal structure, the generation of bile canaliculi, and the activities of CYP enzymes [15]. In HuH‐7 cells, DEX supplementation can restore the functional working of the key hepatic transporters [13, 14] and might also help restore their DMEs functional expression.

This study assessed whether DEX supplementation, in conjunction with an extended cultivation period, could restore compromised hepatic DMEs in HuH‐7 cells. We conducted immunoblotting for major CYPs and uridine 5′‐diphospho‐glucuronosyltransferases (UGTs) and evaluated the metabolic activities of each DME using probe substrates. In addition, we assessed the regulation of DMEs in HuH‐7 cells through nuclear receptors using known inducers and inhibitors.

## 2 Materials and Methods

### 2.1 Cell Culture

The HuH‐7 cells (KCLB60104, Lot No. 48399) were acquired from Korean Cell Line Bank (Seoul, South Korea). The cells were cultivated in T75 flasks (Sarstedt, Newton, NC) in a humidified 5% CO_2_ atmosphere at 37°C. The cells were maintained in high‐glucose Dulbecco's modified Eagle's medium (Gibco 11995‐065; Thermo Fisher Scientific, Waltham, MA) that was supplemented with 10% fetal bovine serum (FBS), penicillin (100 IU/mL), and streptomycin (100 μg/mL). For confluent culture, the cells were seeded (1 × 10^5^ cells/well) on 24‐well plates (353 226; Thermo Fisher Scientific), and they grew to confluence within a few days. During the cultivation for either 1 or 4 week(s), the medium was replaced every 2–3 days. Then, 2 days after seeding, 0, 0.1, or 1 μM DEX was added to the medium. DEX in these concentrations have been reported to improve BSEP expression in HuH‐7 cells [13] and are similar to those widely used for HPH cultures. The expression and activity of DMEs were assessed after 1 or 4 week(s) of culture.

### 2.2 Western Blotting Analysis

The membrane proteins from the cells were prepared using the ProteoExtract Native Membrane Protein Extraction kit (Calbiochem; EMD Biosciences Inc. Darmstadt, Germany). Following treatment of the ligand of nuclear receptors and those for measurement of PXR expression, whole‐cell lysates were harvested using RIPA buffer (1×) with EDTA. Equal amounts (15 μg) of protein were separated using SDS polyacrylamide gel electrophoresis and transferred to polyvinylidene difluoride membranes. The membranes were blocked in Tris‐buffered saline containing 0.1% (v/v) Tween 20 (TBS‐T) with 5% (w/v) fat‐free milk powder and incubated overnight with each primary antibody (anti‐CYP3A4 [ab3572], anti‐CYP1A2 [ab22717], anti‐UGT1A1 [ab237810], anti‐UGT2B7 [ab126269], or anti‐sodium potassium ATPase [ab76020] antibody [Abcam, Cambridge, UK], anti‐CYP1A1 [sc‐25 304] antibody [Santa Cruz Biotechnology, Dallas, TX], anti‐CYP2B6 [TA504328S] antibody [OriGene Technologies Inc., Rockville, MD], or anti‐CYP2C9 [#720237], anti‐PXR [#PA5‐41170], or anti‐beta‐actin [#MA5‐15739] antibody [Invitrogen, Waltham, MA]) in TBS‐T containing 5% (w/v) bovine serum albumin (BSA). After removing the unbound primary antibody with TBS‐T, the membranes were incubated with horseradish peroxidase‐conjugated secondary antibodies (goat anti‐mouse IgG [diluted 1:10000] or goat anti‐rabbit IgG [diluted 1:20000 or 1:10000]; Invitrogen) in TBS‐T containing 5% (w/v) fat‐free milk for 2 h. The signals were detected using Amersham ECL Select Western Blotting Detection Reagent (GE Healthcare, Little Chalfont, UK) on a ChemiDoc XRS imager (Bio‐Rad, Hercules, CA). The signals of the DMEs and nuclear receptors were quantified using ImageJ software and Image Lab 3.0 and were standardized against the Na^+^/K^+^ ATPase and β‐actin signals, respectively.

### 2.3 Measurement of Metabolic Activity

The metabolic activities of DMEs in HuH‐7 cells were assessed using probe substrates of CYPs and UGTs. After aspiration of the medium, the cells were rinsed three times with 300 μL DPBS. Then, the cells were incubated with each probe substrate, namely, 50 μM testosterone (6β‐hydroxylation for CYP3A4 [16]) in DMEM media without any supplement or 20 μM raloxifene (for UGT1A1 [17, 18]) and 100 μM zidovudine (for UGT2B7 [19]) in Hank's balanced salt solution (HBSS) at 37°C in a 5% CO_2_ atmosphere. At each sampling point (1, 2, 4, 8, and 24 h after substrate addition), 50 μL incubation medium was collected from each well, and the same volume of blank medium was replaced. The collected media were stored at −70°C until liquid chromatography–tandem mass spectrometry (LC–MS/MS) analysis of each metabolite.

### 2.4 Inhibition of CYP3A4 and UGT Activities

To evaluate the utility of assessing inhibition according to DME activities in HuH‐7 cells, the accumulation of metabolites in each probe substrate of 50 μM testosterone (6β‐hydroxylation for CYP3A4 [16]), 20 μM raloxifene (for UGT1A1 [17, 18]), and 100 μM zidovudine (for UGT2B7 [19]) was investigated using various concentrations of inhibitors. After aspiration of the medium, the cells were thoroughly rinsed twice with 500 μL DPBS. Then, 400 μL/well DMEM or HBSS was added to prevent cell damage caused by the direct addition of organic solvents. Various concentrations of verapamil (final concentrations of 0, 1, 5, 10, 25, and 50 μM) or ketoconazole (final concentrations of 0, 0.01, 0.05, 0.1, 0.5, and 1 μM) in 2.5 μL 50% methanol were spiked to each well as CYP3A4 inhibitors. For the inhibition of UGT1A1 and UGT2B7, various concentrations of diclofenac (final concentrations of 0, 0.1, 0.5, 1, 5, 10, 50, and 500 μM) in 2.5 μL of 50% DMSO were spiked to each well. To start the reaction, each probe substrate, namely, testosterone (final concentration of 50 μM) in 100 μL DMEM without any supplement or raloxifene and zidovudine (final concentrations of 10 μ and 100 μM, respectively, in the final incubation mixture) in HBSS, was introduced into each well. Following incubation for 4 h in a 5% CO_2_ atmosphere at 37°C, 50 μL incubation medium was collected from each well. The collected medium was stored at −70°C until LC–MS/MS analysis.

### 2.5 LC–MS/MS Analysis of Probe Metabolites

The concentrations of each metabolite in the media were analyzed by LC–MS/MS using an Agilent 6460 triple quadrupole mass spectrometer coupled to an Agilent 1260 LC system (Agilent, Waldbronn, Germany). Instrument control, data acquisition, and processing were performed using Agilent Mass Hunter Workstation software (Version B. 04.01). For the analysis of 6β‐OH‐testosterone, 50 μL medium was mixed with 100 μL methanol containing 1 μg/mL bumetanide as an internal standard (IS). For the analysis of raloxifene glucuronide and zidovudine glucuronide, each 50 μL sample was mixed with 250 μL methanol containing 10 nM OH‐bupropion (IS). Then, the mixture was centrifuged for 5 min at 16000 g, and the supernatant was collected into LC vials. Chromatographic separation was conducted on a reversed‐phase HPLC column (Poroshell 120 C_18_; particle size, 2.7 μm; 3.0 mm i.d. × 50 mm; Agilent), guarded by a SecurityGuard ULTRA column (C18, 4.6 × 50 mm; Phenomenex, Torrance, CA). 6β‐OH‐testosterone and glucuronides of raloxifene and zidovudine were analyzed using isocratic elution of 0.1% formic acid in ultrapure water and methanol (50:50 and 60:40, v/v, respectively) at 0.3 mL/min and 35°C, with run times of 11 and 4.5 min, respectively. The electrospray ionization (ESI) source for the mass spectrometer was used in positive ion mode (ESI^+^) for all analyses. The instrument parameters for the ESI source were set as follows for the analysis of the 6β‐OH‐testosterone and UGTs metabolites, respectively: gas temperature, 270°C and 300°C; sheath gas temperature, 400°C and 250°C; gas flow, 12 and 7 L/min; sheath gas flow, 12 and 11 L/min; and nebulizer, 40 and 45 psi. The capillary voltage was set at 3500 V for both analyses. The fragment voltage, collision energy (CE), *m/z* of precursor and product ions, retention time, and calibration range for each metabolite are presented in Table 1.

**TABLE 1:** LC–MS/MS instrumental parameters, retention times, and calibration ranges for the analysis of metabolites and the respective IS.

Metabolites or IS	Fragmentor voltage (V)	Collision energy (eV)	Precursor ion (*m/z*)	Product ion (*m/z*)	Retention time (min)	Calibration range (μM)
6β‐OH testosterone	135	10	305.1	269.3	3.0	0.02–0.5
Bumetanide (IS for 6β‐OH testosterone)	140	12	365	240.1	9.3	—
Raloxifene‐6‐glucuronide	143	28	650.1	474.2	1.5	0.05–100
Zidovudine glucuronide	95	10	444	127	1.1	5–200
OH‐bupropion (IS for zidovudine glucuronide and raloxifene‐6‐glucuronide)	75	5	256	238	2.2	—

### 2.6 Treatment of the Glucocorticoid Receptor and PXR Inhibitors

To evaluate whether DEX‐induced DME expression in HuH‐7 cells is mediated through glucocorticoid receptor (GR) or PXR, cells were treated with 1 μM DEX alone or co‐treated with the GR inhibitor 1 μM RU‐486 [20] or the PXR inhibitor 2 μM SPA70 [21] from culture day 3 to day 28 and cultured for 4 weeks. Changes in CYP3A4 expression were then assessed by Western blotting analysis described above.

### 2.7 Treatment of PXR, CAR, and Aryl Hydrocarbon Receptor Ligands

To evaluate the regulation of DMEs in HuH‐7 cells, the effects of the treatment of each ligand of nuclear receptors, including PXR, CAR, and aryl hydrocarbon receptor (AhR), on expression of CYPs were studied in HuH‐7 cells cultured for 1 or 4 weeks. To evaluate the responsiveness of HuH‐7 cells to a PXR agonist, rifampicin [22, 23, 24], fresh maintenance medium supplemented with 0, 0.1, or 1 μM DEX with or without 10 μM rifampicin was added on days 25–27 after seeding. To evaluate the effects of the CAR and AhR ligands, the medium was renewed daily with maintenance medium containing either 100 nM CITCO or 50 μM omeprazole, which are well‐known ligands of CAR and AhR, respectively [25, 26], for the last 72 h (on days 4–6 after seeding) of 1‐week HuH‐7 cells. Normal maintenance medium was replaced daily for the controls as these ligands were treated with a very small amount (0.1% [v/v]) of DMSO. After the medium was removed on day 7 or day 28, the cells were rinsed with DPBS. For the Western blotting assay, whole cell lysates were then harvested using 100 μL RIPA Cell Lysis Buffer (1×) with EDTA. Following centrifugation for 15 min (14 000 g, 4°C), the collected cell lysate was kept at −70°C until Western blotting analysis. For the DME gene expression assay, qPCR was performed as described below.

### 2.8 qPCR for a Gene Expression Assay of DMEs

cDNA synthesis from HuH‐7 cultures on 24‐well plates was performed using a FastLane Cell cDNA Kit (Qiagen, Hilden, Germany) according to the manufacturer's instructions. The synthesized cDNA was stored at −20°C. The cDNA stock solution was diluted three times using diethylpyrocarbonate (DEPC) water. The reaction tube for the qPCR was prepared by mixing a diluted cDNA sample (5.5 μL), TaqMan Universal Master Mix (10 μL), DEPC water (3.5 μL), and 1 μL TaqMan Gene Expression Assay probe for CYP3A4 (assay ID: Hs00430021_m1), CYP2B6 (assay ID: Hs04183483_g1), CYP1A1 (assay ID: Hs00153120_m1), CYP1A2 (assay ID: Hs01070369_m1), or GAPDH (assay ID: Hs03929097_g1). qPCR was performed on a CFX96 real‐time system coupled with the C1000TM Thermal Cycler (Bio‐Rad) using CFX Manager management software. The 2^‐ΔΔC^_*T*_ method [27] was used for the data analysis. The target gene was normalized to GAPDH expression.

### 2.9 Data Analysis

Data were analyzed using one‐way analysis of variance (ANOVA) with Tukey's multiple comparisons test or with a nonparametric Kruskal–Wallis test using SPSS 25 Software (IBM Corp., Armonk, NY). Statistical significance was set to *p* < 0.05. Data are shown as mean values with corresponding standard deviations (SDs).

## 3 Results

### 3.1 Effects of DEX on DME Expression and Activities

**FIGURE 1:**
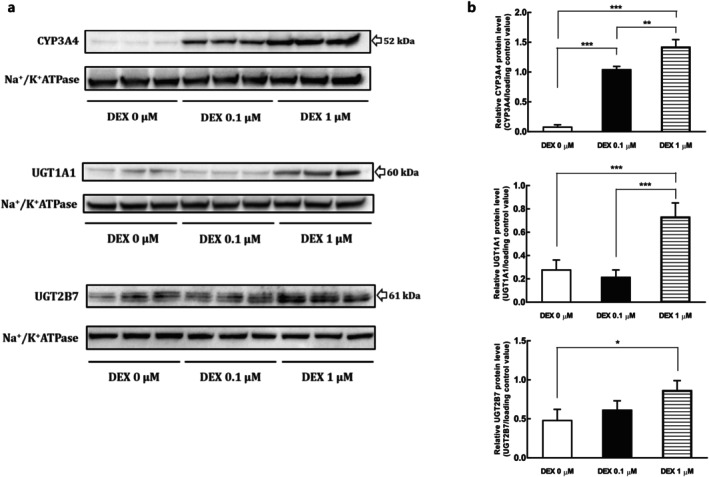
Effect of DEX on the protein levels of CYP3A4, UGT1A1, and UGT2B7 in membrane protein fractions of 4‐week HuH‐7 cells. (a) Representative immunoblots of CYP3A4, UGT1A1, UGT2B7, and Na^+^/K^+^ ATPase (the loading control). (b) Relative expression levels (mean ± SD) of CYP3A4, UGT1A1, and UGT2B7 in 4‐week HuH‐7 cultures with 0, 0.1, and 1 μM DEX treatment from culture day 3 to day 28 (*n* = 3 each). *p < 0.05, **p < 0.01, ***p < 0.001 (one‐way ANOVA with Tukey's test).

**FIGURE 2:**
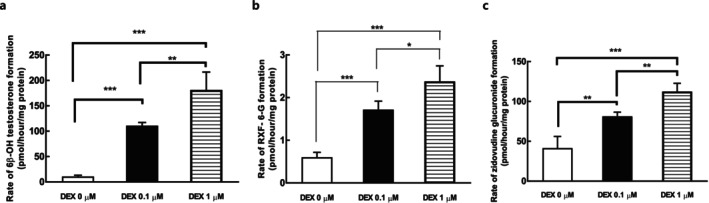
Effect of DEX on the activity of CYP3A4, UGT1A1, and UGT2B7 in 4‐week HuH‐7 cells. Activities (mean ± SD) for (a) 6β‐hydroxylation of testosterone; (b) 6‐glucuronidation of raloxifene (RXF); (c) glucuronidation of zidovudine were measured based on accumulation of metabolites for 4‐h incubation of each substrate (50 μM testosterone, 20 μM raloxifene, or 100 μM zidovudine) with 4‐week HuH‐7 cells with 0, 0.1, and 1 μM DEX treatment from culture day 3 to day 28 (*n* = 4 each). *p < 0.05, **p < 0.01, ***p < 0.001 (one‐way ANOVA with Tukey's test).

The effects of DEX supplementation on the protein levels of DMEs, CYP3A4, UGT1A1, and UGT2B7 in 4‐week confluent HuH‐7 cells were evaluated via immunoblotting (Figure 1). DEX improved the protein expression of these DMEs in HuH‐7 cells. Moreover, the metabolic activities of 6β‐hydroxylation of testosterone and glucuronidation of raloxifene and zidovudine in 4‐week confluent HuH‐7 cells, which are mediated by CYP3A4, UGT1A1, and UGT2B7, respectively, were significantly promoted by DEX treatment (Figure 2). The protein expressions of CYP3A4 and UGT2B7 in HuH‐7 cells were well correlated with the formation of the respective probe metabolites (Supplemental Figure S1). The metabolic activity of UGT1A1 showed a partial lack of correlation with its protein levels, as the protein level remained unchanged while the activity increased at a concentration of 0.1 μM DEX. Separately, we also evaluated effects of DEX on other representative CYPs, and found that DEX supplementation minimally affected the expression levels of CYP1A2, CYP2B6, and CYP2C9 in HuH‐7 cells after 1 and 4 weeks of culture (Supplemental Figure S2).

### 3.2 Inhibition of Metabolic Activities of DMEs

**FIGURE 3:**
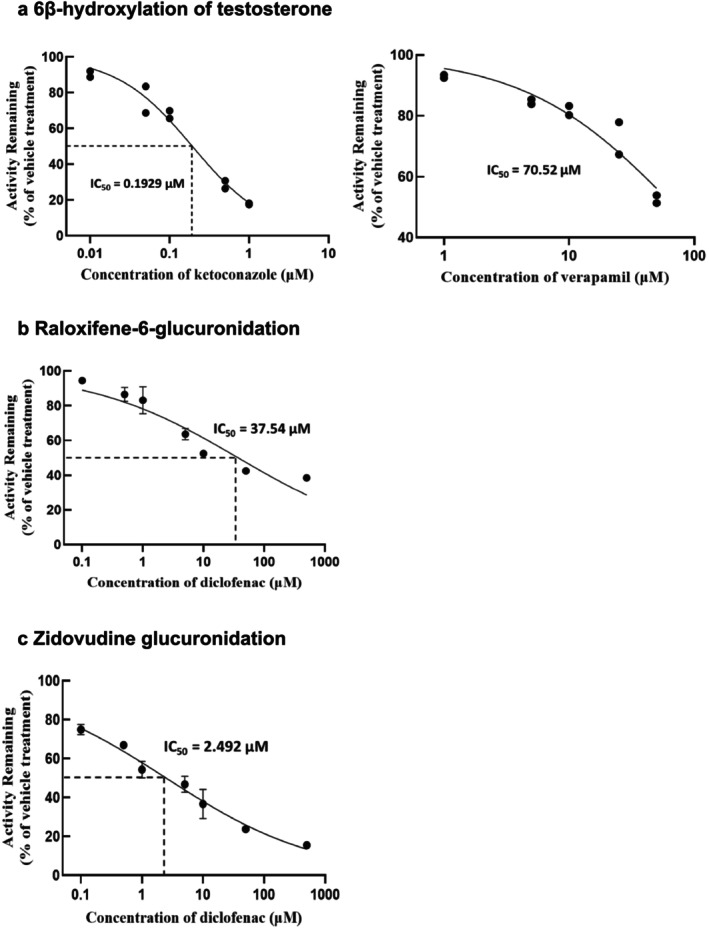
Inhibitory profiles of CYP3A4, UGT1A1, and UGT2B7 mediated metabolism in 4‐week HuH‐7 cells with DEX 1 μM treatment. Remaining activities of (a) 6β‐hydroxylation of testosterone with various concentrations of ketoconazole and verapamil (*n* = 2); (b) 6‐glucuronidation of raloxifene (*n* = 3); and (c) glucuronidation of zidovudine (*n* = 3) with various concentrations of diclofenac in 4‐week HuH‐7 cells with DEX 1 μM treatment. Data were presented as values of duplicate measurements or as mean and SD values.

The metabolic activities in 4‐week confluent HuH‐7 cells promoted by DEX supplementation were further verified using various concentrations of well‐known inhibitors of CYP3A4, UGT1A1, and UGT2B7 (Figure 3). Testosterone 6β‐hydroxylation was inhibited by both ketoconazole and verapamil, CYP3A4 inhibitors, in a concentration‐dependent manner. Raloxifene 6‐glucuronidation (via UGT1A1) and zidovudine glucuronidation (via UGT2B7) were both inhibited by diclofenac. The IC_50_ values for each inhibitor of the testosterone 6β‐hydroxylation, raloxifene 6‐glucuronidation, and zidovudine glucuronidation (via UGT2B7) are summarized in Table 2 with confidence interval (CI).

**TABLE 2:** Inhibitory potency of each inhibitor on testosterone 6β‐hydroxylation, raloxifene 6‐glucuronidation, and zidovudine glucuronidation in 4‐week HuH‐7 cells with DEX 1 μM supplementation.

Metabolic pathway	Inhibitors	IC_50_ (μM)	95% CI	Reported IC_50_ (μM)
Testosterone 6β‐hydroxylation	Ketoconazole	0.1929	0.1595–0.2333	0.22^a^
Testosterone 6β‐hydroxylation	Verapamil	70.52	51.25–117.7	~20^b^
Raloxifene 6‐glucuronidation	Diclofenac	37.54	22.8–67.01	60.9^c^, 57.5^d^
Zidovudine glucuronidation	Diclofenac	2.492	1.952–3.171	3.4^e^, 6.8^f^

^a^ IC_50_ of ketoconazole for 6β‐hydroxylation of testosterone in human liver microsomes (HLM) [28].

^b^ IC_50_ of verapamil for 6β‐hydroxylation of testosterone in HLM [29].

^c,d^ IC_50_ of diclofenac for estradiol 3beta‐glucuronidation in HLM (c) and that for 4‐methylumbelliferone glucuronidation in recombinant human UGT1A1 (d) [30].

^e,f^ IC_50_ of diclofenac for zidovudine glucuronidation in recombinant UGT2B7 (e) and HLM (f) [31].

### 3.3 Effects of GR and PXR Inhibitors on CYP3A4 Expression

**FIGURE 4:**
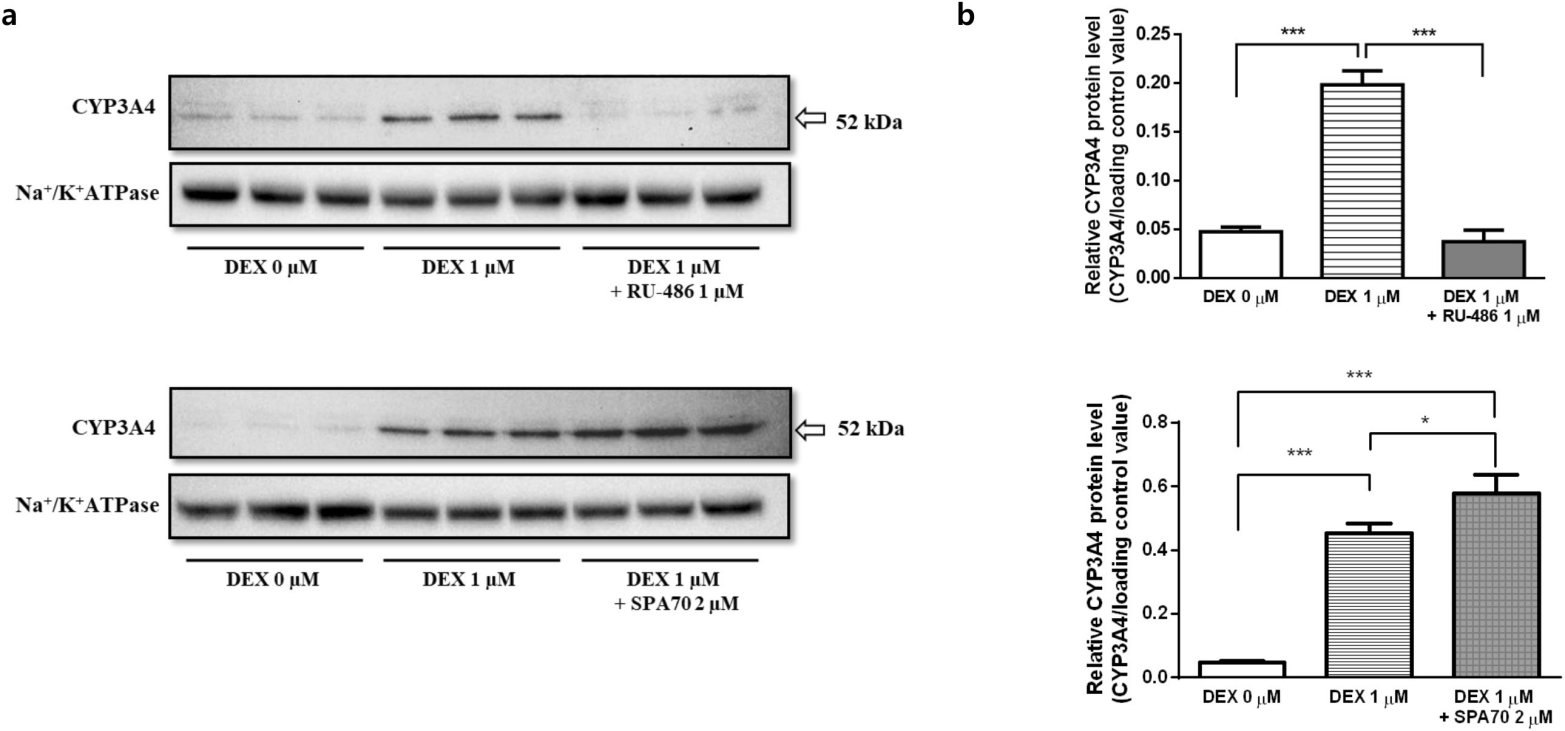
Effect of the GR or PXR inhibitor co‐treatment with DEX on the protein levels of CYP3A4 in membrane protein fractions of 4‐week HuH‐7 cells. (a) Representative immunoblots of CYP3A4 and Na^+^/K^+^ ATPase (the loading control). (b) Relative expression levels (mean ± SD) of CYP3A4 in 4‐week HuH‐7 cultures with 0 and 1 μM DEX treatment with or without co‐treatment of 1 μM RU‐486 (GR inhibitor) and 2 μM SPA70 (PXR inhibitor) from culture day 3 to day 28 (*n* = 3 each). *p < 0.05, ***p < 0.001 (one‐way ANOVA with Tukey's test).

DEX‐induced upregulation of CYP3A4 protein expression in 4‐week confluent HuH‐7 cells was abolished by co‐treatment with the GR inhibitor RU‐486, whereas co‐treatment with the PXR inhibitor SPA70 did not attenuate the induction and instead resulted in a slight further increase (Figure 4). These findings indicate that the DEX‐mediated induction of CYP3A4 occurs primarily through GR activation rather than PXR activation.

### 3.4 Effects of a PXR Ligand on CYP3A4 Expression

**FIGURE 5:**
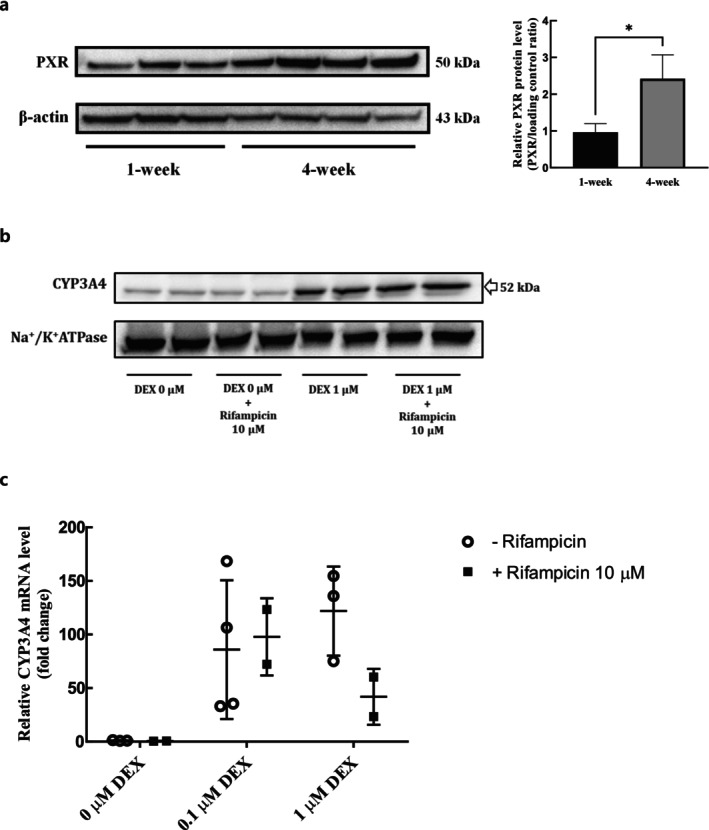
Protein expression of PXR and effects of a PXR ligand on CYP3A4 expression in whole lysates of HuH‐7 cultures. (a) Representative immunoblots and relative expression levels of PXR and β‐actin (the loading control) in 1 and 4‐week HuH‐7 cells. (b) Representative immunoblots of CYP3A4 and Na^+^/K^+^ ATPase (the loading control) with and without 72‐h rifampicin (10 μM) treatment in 4‐week HuH‐7 cells 0 and 1 μM DEX treatment from culture day 3 to day 28. (c) Relative mRNA levels of CYP3A4 with or without 72‐h treatment of 10 μM rifampicin in 4‐week HuH‐7 cells with 0, 0.1, and 1 μM DEX treatment from culture day 3 to day 28. *p < 0.05 (t‐ test).

The protein level of PXR was significantly higher in 4‐week confluent HuH‐7 cells than in 1‐week cells (Figure 5a). However, rifampicin, a PXR ligand, did not induce the protein expression of CYP3A4 in 4‐week confluent HuH‐7 cells, whether supplemented with DEX or not, after 72‐h treatment (Figure 5b). Consistently, mRNA levels of CYP3A4 did not change by 72‐h rifampicin treatment (Figure 5c), regardless of DEX supplementation. By contrast, DEX supplementation itself induced both the transcriptional and translational expression of CYP3A4.

### 3.5 Effects of a CAR Ligand and an AhR Ligand in 1‐Week Confluent HuH‐7 Cells

**FIGURE 6:**
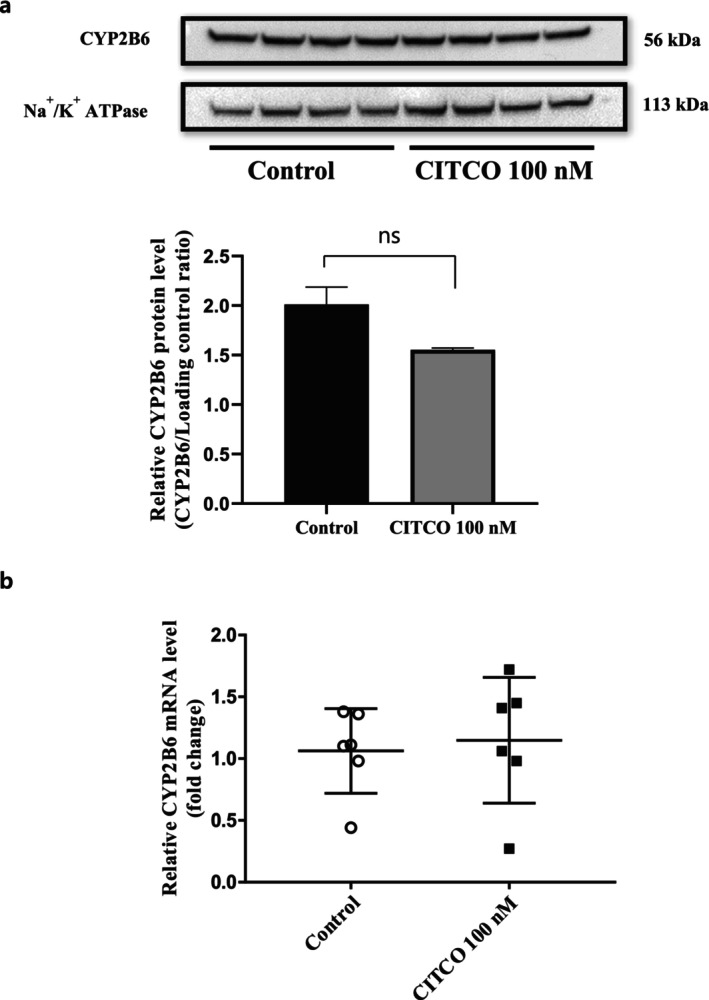
Effects of a CAR ligand on CYP2B6 expression in whole lysates of HuH‐7 cultures. (a) Representative immunoblots and relative expression levels of CYP2B6 and Na^+^/K^+^ ATPase (the loading control) in 1‐week HuH‐7 cultures with and without 72 h CITCO (100 nM) treatment. (b) Relative mRNA levels of CYP2B6 with or without 72‐h treatment of 100 nM CITCO in 1‐week HuH‐7 cultures.

**FIGURE 7:**
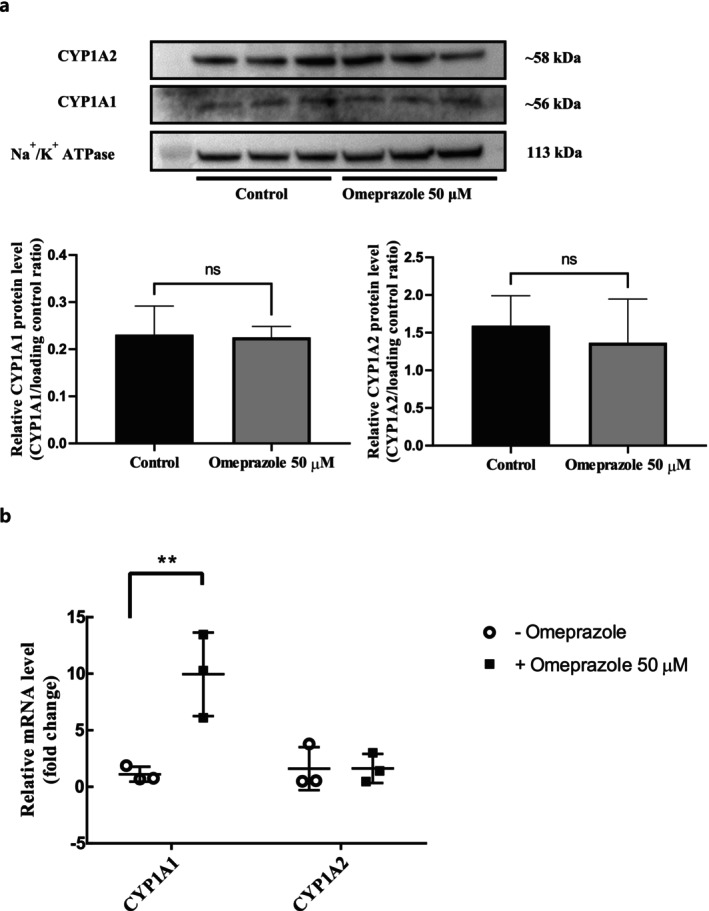
Effects of a AhR ligand on CYP1A1/2 expression in whole lysates of HuH‐7 cultures. (a) Representative immunoblots and relative expression levels of CYP1A1/2 and Na^+^/K^+^ ATPase (the loading control) in 1‐week HuH‐7 cultures with and without 72 h omeprazole (50 μM) treatment. (b) Relative mRNA levels of CYP1A1/2 with or without 72‐h treatment of 50 μM omeprazole in 1‐week HuH‐7 cultures.

The protein expression and mRNA level of CYP2B6 in 1‐week confluent HuH‐7 cells were not influenced by the 72‐h treatment with a CAR ligand (100 nM CITCO) (Figure 6). Treatment with an AhR ligand (50 μM omeprazole) for 72 h significantly induced CYP1A1 mRNA expression without affecting its protein level in 1‐week confluent HuH‐7 cells, while both the mRNA and protein levels of CYP1A2 remained unchanged (Figure 7).

## 4 Discussion

Hepatic cell lines fail to express sufficient levels of DMEs or transporters to predict hepatic drug disposition, although they offer better consistency and affordability to utilize than HPHs [2, 4, 9]. It has recently been reported that maintaining HuH‐7 cells at confluency over several weeks with additional supplements, such as DEX and/or DMSO, promotes some hepatic CYP isozymes and transporters' expression [10, 13, 14]. An extracellular matrix overlay (Matrigel) of HuH‐7 cells restores the adequate localization and activity of principal ABC transporters in canaliculi such as BSEP and MDR1 [13]. However, the effects of modified culture methods on the levels of key hepatic DMEs and their regulation in HuH‐7 cells have yet to be evaluated. This study focused on investigating whether glucocorticoid treatment (DEX), a key component of HPH cultures, could ameliorate the expression level and metabolic activity of key DMEs (CYPs and UGTs) in confluent HuH‐7 cells. In general, DEX treatment promoted the protein level and activity of CYP3A4 and UGTs. However, the transcriptional regulation of DMEs through the nuclear receptors, including PXR and CAR, did not work properly in HuH‐7 cells.

First, DEX supplementation in culture media significantly induced the protein level and metabolic activity of CYP3A4, UGT1A1, and UGT2B7 in 4‐week confluent HuH‐7 cells in a concentration‐dependent manner. This is consistent with a recent report of an increase in the transcription of CYP3A4 in Matrigel‐overlaid 2‐week confluent HuH‐7 cells with DEX supplementation [14]. In that study, the protein level of UGT2B7 was not affected by DEX supplementation, which is not consistent with our results. This difference may be due to differences in culture period or the presence or absence of Matrigel overlay. DEX is essentially applied at nanomolar concentrations (25–100 nM) in HPHs culture to retain specific hepatic cell functions [32]. In HuH‐7 cells, transcriptional regulation of DMEs via PXR or CAR was not observed based on our results. This may be due to low expression or dysfunction of these nuclear receptor and/or cofactors and signaling pathways required for full receptor activation. Therefore, the observed DEX concentration‐dependent increases in the expression and activity of CYP3A4, UGT1A1, and UGT2B7 are likely primarily mediated through GR. Our findings (Figure 4) further support that the inductive effects of DEX on DMEs are predominantly GR‐dependent rather than mediated through PXR. DEX‐induced increase in CYP3A4 expression was abolished by co‐treatment with the GR inhibitor RU‐486, whereas SPA70, a selective PXR inhibitor, failed to reduce this induction and instead produced a slight further increase. While GR‐mediated induction of CYP3A4 and UGT1A1 has been well‐documented [33, 34], the involvement of GR in the regulation of UGT2B7 expression has not yet been demonstrated.

Notably, DEX‐enhanced metabolic activities in 4‐week confluent HuH‐7 cells enabled reliable assessment of inhibition using established reference inhibitors. Therefore, the improved metabolic activities of these DMEs in modified HuH‐7 cultures could be applicable for evaluating the inhibition potential of test compounds. While modified HuH‐7 cultures are not as efficient as human liver microsomes for the evaluation of DME inhibition, they could serve as a surrogate live cell model featuring major DME activities and can be used for long‐term in vitro evaluation of hepatotoxicity and relevant mechanisms. The IC_50_ value of ketoconazole for CYP3A4‐mediated 6β‐hydroxylation of testosterone in the human liver microsome is 0.22 μM [28], similar to the value obtained in our modified HuH‐7 cells. Verapamil, a moderate mechanism‐based inhibitor, showed a higher IC_50_ value (~20 μM) for the 6β‐hydroxylation of testosterone [29] than the potent inhibitor ketoconazole, which is also consistent with the result obtained in our modified 4‐week confluent HuH‐7 cultures. Diclofenac exhibited a lower IC₅₀ value for UGT2B7 than for UGT1A1, with values comparable to those previously reported [30, 31].

The activities of other CYP isozymes (CYP2B6 and 2C9) tended to increase slightly in response to 1 μM DEX supplementation in 4‐week HuH‐7 cultures (Supplemental Figure S3). However, the protein levels of CYP1A2, CYP2B6, and CYP2C9 were not significantly influenced by DEX supplementation or the culturing period. The minimal effect of DEX on the expression of these CYP isozymes is consistent with the results observed in HPHs [35, 36, 37]. Although the expression of these CYPs, with the exception of CYP3A4, was not significantly promoted by the modification of the culture conditions, it was confirmed that HuH‐7 cells expressed these CYPs to a certain level and also had metabolic activity.

To avoid the possibility of DDI in clinical candidate compound selection, it is important to evaluate CYP3A4, CYP2B6, and 1A1/2 induction via the activation of NRs, including PXR, CAR, and AhR, that regulate transcription of major hepatic DMEs [38, 39]. HPHs are largely used to evaluate the induction potential of investigational drugs by monitoring the mRNA levels or activities of DMEs. Therefore, the regulation of DMEs through nuclear receptors was further assessed in HuH‐7 cells.

The expression of PXR in HuH‐7 cells was significantly enhanced using an extended culturing period (Figure 5a), which is consistent with a previous report that indicated that long‐term confluent culture of HuH‐7 cells induced gene expression of PXR [12]. However, the induction of CYP3A4 via a PXR ligand, rifampicin [40], was not successful in 4‐week HuH‐7 cells irrespective of DEX supplementation. Although it has been hypothesized that DEX supplementation may further induce PXR expression based on previous reports [35, 40, 41], the protein expression of PXR was not additionally promoted by DEX supplementation in 4‐week confluent HuH‐7 cells (Supplemental Figure S4). Therefore, further studies are needed to determine why a PXR ligand failed to induce CYP3A4 even in 4‐week confluent HuH‐7 cells, where the protein expression of PXR was promoted. The transcription of CYP3A4 by PXR activation requires other coactivators, such as HNF4α, PGC1α, SRC1, CBP, and P300 [42, 43]. Therefore, the level of these coactivators should be further verified in HuH‐7 cells. The possible lower expression of uptake transporters involving rifampicin uptake, such as OATP1B1/1B3 [9], could be the reason for the unresponsiveness of HuH‐7 cells to rifampicin treatment. In addition, 1‐week confluent HuH‐7 cells did not exhibit inducibility of CYP2B6 expression by CITCO, a CAR ligand. These findings are in line with prior research by Jouan et al. [9], which indicated a lack of response of hepatic drug transporters to the prototypical activators of PXR (rifampicin) and CAR (phenobarbital) in HuH‐7 cells.

By contrast, transcription of CYP1A1 in 1‐week confluent HuH‐7 cells was induced by omeprazole (an AhR ligand). Our findings align with prior research that indicated an induction of breast cancer resistance protein (BCRP) following the exposure of HuH‐7 cells to a prototypical ligand of AhR (2,3,7,8‐tetrachlorodibenzo‐*p*‐dioxin, TCDD) [9]. However, the protein level of CYP1A1, as well as both the mRNA and protein levels of CYP1A2, were not induced by the omeprazole treatment in 1‐week confluent HuH‐7 cells.

## 5 Conclusions

In conclusion, the metabolic activities of DMEs such as CYP3A4, UGT1A1, and UGT2B7 in HuH‐7 cells were activated by DEX possibly through GR activation and extended culturing periods in confluency; the levels of induction were sufficient for inhibition studies. However, HuH‐7 cells failed to demonstrate the normal transcriptional regulation of CYPs after PXR or CAR activation. Only the transcriptional induction of CYP1A1 via the activation of AhR functioned in 1‐week confluent HuH‐7 cultures. Further studies are required for additional characterization and improvement of HuH‐7 cells to serve as a reliable in vitro hepatic model.

## Author Contributions

Hana Yu and Song Hee Lee conducted the experiments, data curation, formal analysis, and writing – original draft. Ji Hyeon Kim and Seung Jin Kim performed the experiments and data analysis. Hee Eun Kang was responsible for conceptualization, methodology, data curation, formal analysis, and writing the manuscript.

## Funding

This research received support from the National Research Foundation of Korea (NRF), funded by the Korean Government Ministry of Education (No. 2018R1A6A1A03025108) and MSIT (No. 2019R1F1A1052243).

## Conflicts of Interest

The authors declare no conflicts of interest.

## Supporting information


**Figure S1:** **Correlation between expression and activity of CYP3A4 and UGT2B7.** Pearson correlation analysis between protein expression levels of CYP3A4 (A) and UGT2B7 (B) and their metabolic activities measured by metabolite formation rate from each probe substrate in 4‐week HuH‐7 cultures supplemented with DEX 0, 0.1, and 1 μM (*n* = 3 each) from culture day 3 to day 28.
**Figure S2: Effects of DEX on protein expression of CYP3A4, CYP1A2, CYP2B6, and CYP2C9 in 1‐ and 4‐week HuH‐7 cultures.** Representative immunoblots of CYP3A4, CYP1A2, CYP2B6, CYP2C9, and Na^+^/K^+^ ATPase (the loading control in 1‐ and 4‐week HuH‐7 cultures supplemented with 0, 0.1, and 1 μM DEX from culture day 3 to day 7 or to day 28). The same amount of protein (10 μg) prepared by membrane protein extraction was loaded into each lane.
**Figure S3: Metabolic activity of CYP2B6 and 2C9 in HuH‐7.** Effects of DEX supplementation on metabolic activities of CYP2B6 and CYP2C9 in 4‐week HuH‐7 cultures. Accumulation of OH‐bupropion (A) and 4′‐OH‐diclofenac (B) in incubation media (DMEM media without other supplements) containing cocktail probe substrates (50 μM bupropion and 10 μM diclofenac) were measured for 24 h (*n =* 2*,* each).
**Figure S4: Effects of DEX on protein expression of PXR in 4‐week HuH‐7 cultures.** Representative immunoblots of PXR and β‐actin (the loading control) in 4‐week HuH‐7 cultures supplemented with 0, 0.1, and 1 μM DEX (*n* = 3 each). The same amount of protein (20 μg) prepared by whole cell lysis was loaded into each lane.

## Data Availability

The corresponding author will provide original raw data upon request.
