# Supplementary material for: Dexamethasone‐Mediated Regulation of CYP3A4 and UGTs in Human Hepatoma HuH‐7 Cells
Source: Fundam Clin Pharmacol. 2026 Apr 21;40:e70089. doi: 10.1111/fcp.70089 (PMC13099280; doi:10.1111/fcp.70089)
Supplement: Supplementary file 1 — Figure S1: Correlation between expression and activity of CYP3A4 and UGT2B7. Pearson correlation analysis between protein expression levels of CYP3A4 (A) and UGT2B7 (B) and their metabolic activities measured by metabolite formation rate from each probe substrate in 4‐week HuH‐7 cultures supplemented with DEX 0, 0.1, and 1 μM (n = 3 each) from culture day 3 to day 28. Figure S2: Effects of DEX on protein expression of CYP3A4, CYP1A2, CYP2B6, and CYP2C9 in 1‐ and 4‐week HuH‐7 cultures. Representative immunoblots of CYP3A4, CYP1A2, CYP2B6, CYP2C9, and Na+/K+ ATPase (the loading control in 1‐ and 4‐week HuH‐7 cultures supplemented with 0, 0.1, and 1 μM DEX from culture day 3 to day 7 or to day 28). The same amount of protein (10 μg) prepared by membrane protein extraction was loaded into each lane. Figure S3: Metabolic activity of CYP2B6 and 2C9 in HuH‐7. Effects of DEX supplementation on metabolic activities of CYP2B6 and CYP2C9 in 4‐week HuH‐7 cultures. Accumulation of OH‐bupropion (A) and 4′‐OH‐diclofenac (B) in incubation media (DMEM media without other supplements) containing cocktail probe substrates (50 μM bupropion and 10 μM diclofenac) were measured for 24 h (n = 2, each). Figure S4: Effects of DEX on protein expression of PXR in 4‐week HuH‐7 cultures. Representative immunoblots of PXR and β‐actin (the loading control) in 4‐week HuH‐7 cultures supplemented with 0, 0.1, and 1 μM DEX (n = 3 each). The same amount of protein (20 μg) prepared by whole cell lysis was loaded into each lane. [file FCP-40-0-s001.docx]

**A**

**B**

**Supplemental Fig. 1. Correlation between expression and activity of CYP3A4 and UGT2B7.** Pearson correlation analysis between protein expression levels of CYP3A4 (A) and UGT2B7 (B) and their metabolic activities measured by metabolite formation rate from each probe substrate in 4-week HuH-7 cultures supplemented with DEX 0, 0.1 and 1 μM (*n* = 3 each) from culture day 3 to day 28.

**Na^+^/K^+^ ATPase**


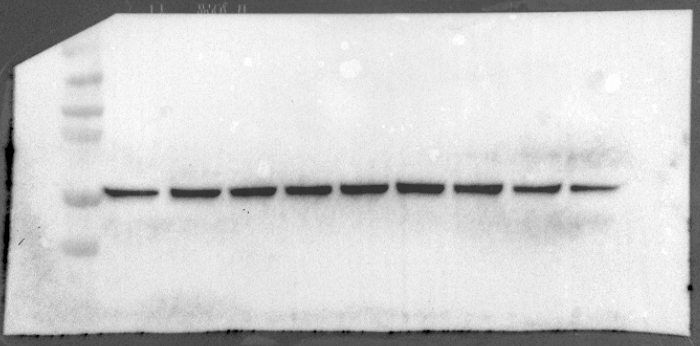


**CYP2B6**


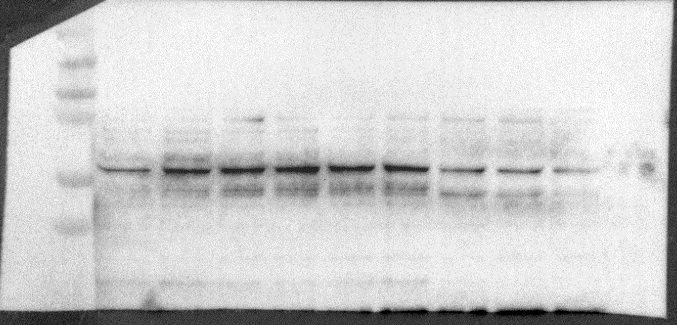


**CYP2C9**

**56 kDa**

**56 kDa**


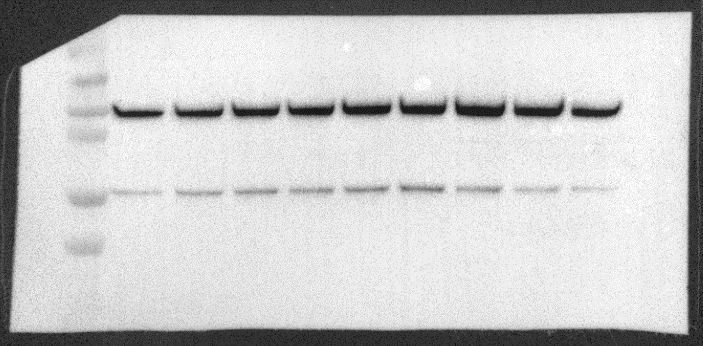


**113 kDa**

**DEX 0 μM**

**DEX 0.1 μM**

**DEX 1 μM**

**1 week**

**4 week**

**DEX 0, 0.1, 1 μM**


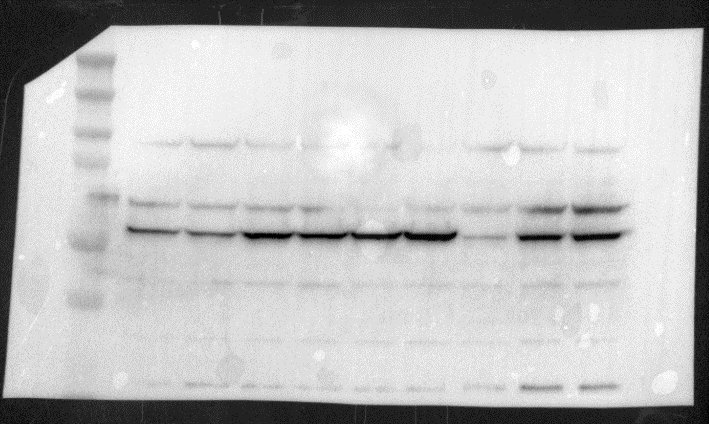


**CYP3A4**

**52 kDa**


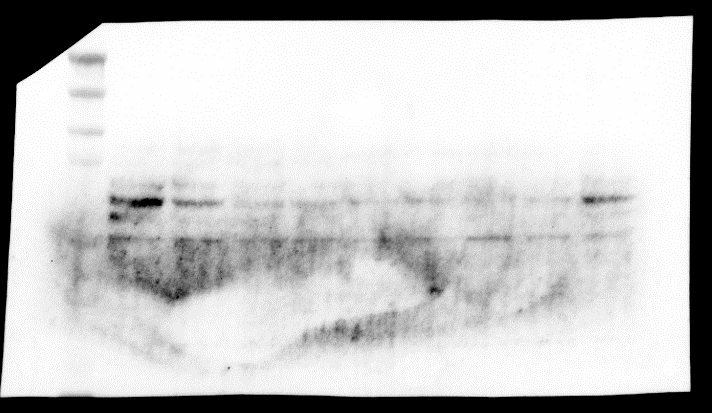


**~58 kDa**

**CYP1A2**


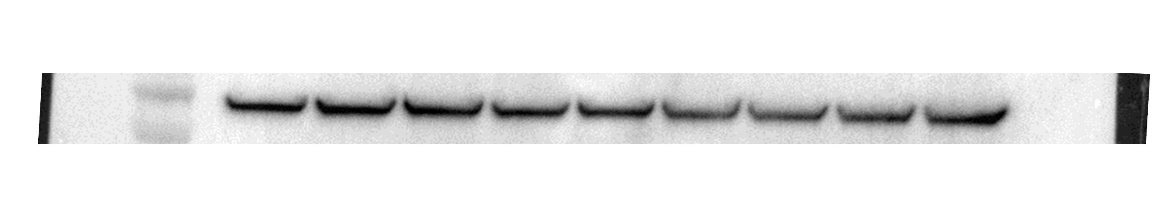


**113 kDa**

**Na^+^/K^+^ ATPase**

**Supplemental Fig. 2. Effects of DEX on protein expression of CYP3A4, CYP1A2, CYP2B6, and CYP2C9 in 1- and 4-week HuH-7 cultures.** Representative immunoblots of CYP3A4, CYP1A2, CYP2B6, CYP2C9 and Na^+^/K^+^ ATPase (the loading control in 1 & 4-week HuH-7 cultures supplemented with 0, 0.1, and 1 μM DEX from culture day 3 to day 7 or to day 28. The same amount of protein (10 μg) prepared by membrane protein extraction was loaded into each lane.

**A B**

**Supplemental Fig. 3. Metabolic Activity of CYP2B6 and 2C9 in HuH-7.** Effects of DEX supplementation on metabolic activities of CYP2B6 and CYP2C9 in 4-week HuH-7 cultures. Accumulation of OH-bupropion (A) and 4’-OH-diclofenac (B) in incubation media (DMEM media without other supplements) containing cocktail probe substrates (50 μM bupropion and 10 μM diclofenac) were measured for 24 h (*n =* 2*,* each).

**Supplemental Fig. 4. Effects of DEX on protein expression of PXR in 4-week HuH-7 cultures.** Representative immunoblots of PXR and β-actin (the loading control) in 4-week HuH-7 cultures supplemented with 0, 0.1, and 1 μM DEX (*n* = 3 each). The same amount of protein (20 μg) prepared by whole cell lysis was loaded into each lane.
